# Anti-methanogenic effect of phytogenic extract of *Moringa oleifera* on methane mitigation through inhibition of methyl-coenzyme M reductase receptor: *In silico* study

**DOI:** 10.5455/javar.2025.l904

**Published:** 2025-04-30

**Authors:** Muhammad Sulaiman Daulai, Indah Wijayanti, Yuli Retnani, Suzuki Toshisada

**Affiliations:** 1 Graduate School of Nutrition and Feed Science, Faculty of Animal Science, IPB University, Bogor, Indonesia; 2 Department of Animal Nutrition and Feed Technology, Faculty of Animal Science, IPB University, Bogor, Indonesia; 3 Department of Biological Molecular Chemistry, Faculty of Agriculture, Kagawa University, Kagawa, Japan

**Keywords:** methyl-coenzyme M reductase, *Moringa oleifera* L., phytogenic extract

## Abstract

**Objectives:**

This study aimed to assess the anti-methanogenic potential of *Moringa oleifera* L. phytogenic extracts through *in silico* inhibition of the methyl-coenzyme M reductase (MCR) receptor.

**Materials and Methods:**

Phytochemicals from *M*. *oleifera* were screened and compared with anti-methanogenic compounds such as 3-nitrooxypropanol and native MCR enzyme ligands (coenzyme M and coenzyme B). Molecular docking analysis was performed using AutoDock Vina on PyRx 0.8, and interactions were visualized with Discovery Studio 2024.

**Results:**

Selected phytochemicals, including pterygospermin, exhibited promising drug-likeness based on Lipinski’s rule of five and absorption, distribution, metabolism, excretion, and toxicology properties. Pterygospermin demonstrated the highest binding affinity to the MCR enzyme’s active site, with interactions including Pi-sulfur (Phe443), Pi-alkyl (Val482, Leu320, and Met324), Pi-Pi stacking (Phe330), and van der Waals forces (Tyr333 and Ser325).

**Conclusion:**

Pterygospermin shows potential as a competitive inhibitor of the MCR enzyme, providing a sustainable approach to mitigate methane emissions in livestock and contribute to global greenhouse gas reduction efforts.

## Introduction

The livestock industry contributes significantly to greenhouse gas emissions worldwide, primarily through methane (CH4) production from enteric fermentation in ruminants, which accounts for approximately 88% of the sector’s emissions globally [1]. Methane emissions represent an inefficient utilization of feed energy, as a portion of the feed is converted into CH4 and released into the atmosphere, leading to economic losses for farmers and contributing to climate change. With the global demand for animal-derived products projected to double by 2050, driven by increasing living standards, mitigating methane emissions is imperative to enhance livestock productivity and minimize the industry’s environmental impact [2]. However, various studies of methane mitigation have been implemented; the anticipated long-term reductions have not been realized. Recently, the majority of approaches to reducing enteric methane emissions have centered on diet and feed additives [3]. In tropical regions, ruminants may benefit significantly from the protein supplements provided by the plant materials, which improve nutritional digestibility and reduce methane emissions. Among these, *Moringa oleifera* stands out due to its widespread availability in tropical and subtropical climates, rapid growth, and high biomass yield. Furthermore, research has demonstrated that dietary supplementation with *M. oleifera* optimizes microbial metabolic functions and reduces methane emissions. Vitamins, selenium, flavonoids, phenolics, and carotenoids are all abundant in *M. oleifera* leaves and seed extract, making them a nutritious and healthful substance that can help reduce CH_4_ emissions [4].

Identifying key compounds and phytochemicals in *M. oleifera* with potential anti-methanogenic properties is essential. Many plants have been thoroughly researched for their diverse biological activities, and the phytochemicals extracted from *M. oleifera* can be compared with other anti-methanogenic compounds to assess their ability to inhibit critical enzymes involved in methane production in ruminants. It is important to recognize that each plant contains a range of phytochemicals, each with distinct functions and varying impacts on methane emissions. The limited application of computational screening techniques to identify natural inhibitors of methane production has slowed progress, especially since plants like *M. oleifera* harbor multiple phytochemicals. Advanced computational methods allow for the efficient evaluation of various compounds, overcoming the limitations of labor-intensive *in vitro* studies and accelerating the discovery process. *In silico* studies, therefore, present an opportunity to screen numerous phytochemicals against CH4 emissions, potentially identifying effective inhibitors without the need for *in vitro* labor resources [5].

Molecular docking stands as a commonly employed method for *in silico* screening, allowing biomolecules to interact with a target receptor. Therefore, this study focuses on screening compounds to identify prospective inhibitors of the methyl-coenzyme M reductase (MCR) receptor. The MCR enzyme is necessary for methanogenesis in rumen archaea bacteria. As a result, the MCR protein is commonly used in a variety of applications as a methanogenesis marker. Inhibiting MCR can suppress the activity of ruminal methanogens, thereby reducing enteric methane emissions in ruminants [6]. Those phytochemicals showing promise in targeting the enzyme could be candidates for subsequent *in vitro* studies and could be developed as phytogenic feed additives for ruminants. These natural compounds could reduce methane emissions from livestock, thereby lowering the sector’s overall greenhouse gas footprint, contributing to sustainable agricultural practices, and fostering climate resilience. The adoption of such eco-friendly solutions could pave the way for more sustainable systems while reducing reliance on synthetic chemicals, offering both environmental and economic benefits. This study aimed to assess the anti-methanogenic potential of *M. oleifera* L. phytogenic extracts through *in silico* inhibition of the MCR.

## Material and Methods

### Ligands selection

**Figure 1.**
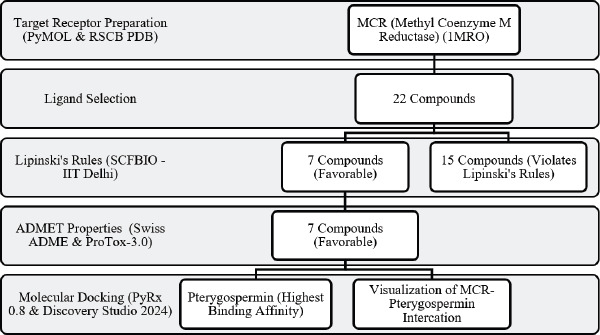
Workflow diagram of *in silico* study. Adapted with the help of Liu et al. [10].

A total of 22 phytochemicals, which are exclusively derived from the leaves and seeds of *M. oleifera*, were selected from the literature database according to the prior reports by the PubChem database and will be compared with another anti-methanogenic compound [3-nitrooxypropanol (NOP)] and native ligands [coenzyme M (CoM) & coenzyme B (CoB)]. All phytochemical structures were obtained in structured data file and Protein Data Bank (PDB) format from PubChem (pubchem.ncbi.nlm.nih.gov). A workflow of the methodology is presented in Figure 1.

### Lipinski’s rule of five

Lipinski’s rules were used to assess each ligand’s drug-likeness (scfbio-iitd.res.in/software/drugdesign/lipinski.jsp). This rule assesses the possible drug candidate’s stability as well as important characteristics such as molecular weight, logP, the number of hydrogen bond donors and acceptors, and molar refractivity [7].

### Absorption, distribution, metabolism, excretion, and toxicology (ADMET) properties analysis

The Swiss ADME tool from the Swiss Institute of Bioinformatics (swissadme.ch) was used to examine the ADMET properties of ligands that satisfy Lipinski’s rule of five. The program was used to evaluate the canonical simplified molecular input line entry system, which was acquired from PubChem. Water solubility (log mol/l), lipophilicity (log Po/w), blood-brain barrier (BBB) permeability, gastrointestinal (GI) absorption, and P-gp substrate characteristics were among the attributes that the tool assessed. The Swiss ADME tool utilizes a vector machine algorithm, enabling efficient analysis of datasets for known inhibitors/noninhibitors and substrates/nonsubstrates [8]. Additionally, the evaluation of oral toxicity employed the ProTox 3.0 tool (tox.charite.de/protox3) to predict the lethal dose 50 (LD50) value of compounds. The toxicity results that emerged were then analyzed for acute oral toxicity categories, allowing for a robust preliminary assessment of the toxicological risks associated with oral exposure to the compounds under study [9]. Selected phytochemicals were used for molecular docking analysis.

### Target receptor of study

The RCSB PDB—1MRO (rscb.org/pdb) provided the 3D structure of MCR. PyMOL was used to eliminate any unnecessary water molecules and inhibitors that were attached to the receptor during docking. Energy minimization was performed on both the MCR receptor and the chosen ligands before docking.

### Molecular docking

Phytochemical compounds that did not violate Lipinski’s rule of five and exhibited moderate “drug-likeness” were chosen for the molecular docking process. Protein-ligand docking was employed in this study, with MCR as the target receptor. In this study, the docking grid coordinates were carefully chosen to ensure the accuracy and relevance of the *in silico* molecular docking analysis. The docking grid box was centered at coordinates *x* = 24.7829, *y* = 36.2113, and *z* = −12.9281 to align with the active site of the MCR enzyme. These coordinates correspond to the region where the native ligands, CoM, and CoB, typically bind during the enzymatic process that produces methane in ruminants. The root means square deviation (RMSD) of 0.149 Å was also used as a benchmark for comparing the alignment of docked ligands with the native ligand’s conformation, ensuring the reliability of the results. Molecular docking was employed to evaluate the binding affinity and interaction dynamics between phytochemical compounds and the MCR target receptor, which is the crucial enzyme in the rumen that catalyzes methane production, by analyzing the binding energy of the drug–protein complex. AutoDock Vina 1.5.6 from PyRx 0.8 (Virtual Screening Tools) was employed for this analysis, and the results were visualized using the BIOVIA Discovery Studio 2024 tool.

## Results

### Ligands selection

This study revealed that selected ligands in Table 1, classified as selected ligands that were exclusively derived from *M. oleifera*, were evaluated using the PubChem database and relevant literature from Liu et al. [10]. This finding compared selected ligands to comparative ligands (3-NOP) and native ligands, which are substrates of MCR (CoM & CoB).

**Table 1.** Selected compound of **M. oleifera*,* comparative ligands, and native ligands through virtual screening.

No	Compound	PubChem ID	Sources
Moringa oleifera compound/selected ligand			
1	Quercetin-3'-glucoside	9934142	Seeds, leaves
2	Niazinin	10088810	Leaves, seeds
3	Niazimicin A	10247749	Leaves, seeds
4	O-Methyl-4-[(2',3',4'-tri-O-acetyl-alpha-L-rhamnosyloxy) benzyl]carbamate	101919834	Leaves
5	O-Ethyl-4-[(2',3',4'-tri-O-acetyl-alpha-L-rhamnosyloxy)benzyl] carbamate	10434741	Leaves
6	Marumoside A	101794623	Leaves
7	Pterygospermin	72201063	Seeds, leaves
8	Moringyne	131751186	Seeds
9	4-Caffeoylquinic acid;4-O-Caffeoylquinic acid	58427569	Leaves
10	Niazirinin	10426197	Leaves, seeds
11	Niazirin	129556	Seeds, leaves
12	4-[(4'-O-Acetyl-alpha-L-rhamnosyloxy)benzyl]isothiocyanate	10291650	Seeds
13	Glucomoringin	162639104	Seeds
14	Glucosinalbin	9601115	Seeds
15	Glucoraphanin	6602383	Seeds
16	Glucoiberin	9548622	Seeds
17	Benzyl glucosinolate	21600402	Seeds
18	Glucotropaeolin	9548605	Seeds
19	Glucobarbarin	138756720	Seeds
20	Glucoraphenin	656559	Seeds
21	Niazimin	10339912	Leaves, seeds
22	Niazicinin A	101920262	Leaves, seeds
Comparative ligand			
23	3-NOP	10011893	
Native ligand			
24	CoM & CoB		

### Moringa oleifera compound’s drug-likeness properties

The drug-likeness properties of *M. oleifera* were predicted by Lipinski’s rule of five. The drug-likeness properties analysis determined the physicochemical characteristics of compounds, including their permeability or ability to diffuse through cell membranes [11]. This result, as shown in Table 2, presents the molar refractivity, log P, number of hydrogen bond donors, number of acceptors, and molecular weight of the selected compounds (Table 2). In line with Lipinski’s rule of five, seven molecules were chosen as the best ligands since they satisfied every requirement: 4-[(4’-O-Acetyl-alpha-L-rhamnosyloxy)benzyl]isothiocyanate, niazinin, niazicinin A, pterygospermin, niazirinin, and niazimin.

**Table 2.** Selected compounds of **M. oleifera* were* analyzed by Lipinski’s rule.

No	Compound	Molecular weight (Da)	H acceptor	H donor	Log P	Molar refractivity
1	Quercetin-3'-glucoside	464.38	12	8	−0.54	110.16
2	Niazinin	343.40	6	4	0.3	85.68
3	Niazimicin A	357.42	6	4	0.69	90.49
4	O-Methyl-4-[(2',3',4'-tri-O-acetyl-alphaL-rhamnosyloxy)benzyl]carbamate	453.44	10	1	1.85	107.69
5	O-Ethyl-4-[(2',3',4'-tri-O-acetyl-alpha-L-rhamnosyloxy)benzyl]carbamate	467.47	10	1	2.24	112.50
6	Marumoside A	297.30	6	4	−0.38	72.11
7	Pterygospermin	406.52	2	0	4.02	123.32
8	Moringyne	312.32	7	4	−0.74	75.31
9	4-Caffeoylquinic acid;4-O-Caffeoylquinic acid	354.31	9	6	−0.65	83.5
10	Niazirinin	321.33	7	2	0.53	78.68
11	Niazirin	279.29	6	3	−0.04	68.95
12	4-[(4'-O-Acetyl-alpha-L-rhamnosyloxy) benzyl]isothiocyanate	353.39	7	2	1.07	88.1
13	Glucomoringin	609.66	15	7	−2.16	122.7
14	Glucosinalbin	425.43	11	6	−0.32	93.6
15	Glucoraphanin	437.51	11	5	0.15	94.59
16	Glucoiberin	423.48	11	5	−0.24	89.78
17	Benzyl glucosinolate	408.42	10	4	−0.37	89.72
18	Glucotropaeolin	409.43	10	5	−0.02	91.57
19	Glucobarbarin	477.55	11	5	−0.49	95.69
20	Glucoraphenin	435.49	11	5	0.27	94.12
21	Niazimin	383.39	8	3	1.1	93.03
22	Niazicinin A	369.37	8	3	0.71	88.22

Description: = violates Lipinski’s rules; = negative value of Log P; = favorable compound by Lipinski’s rule.

### ADMET properties of M. oleifera compounds

**Figure 2.**
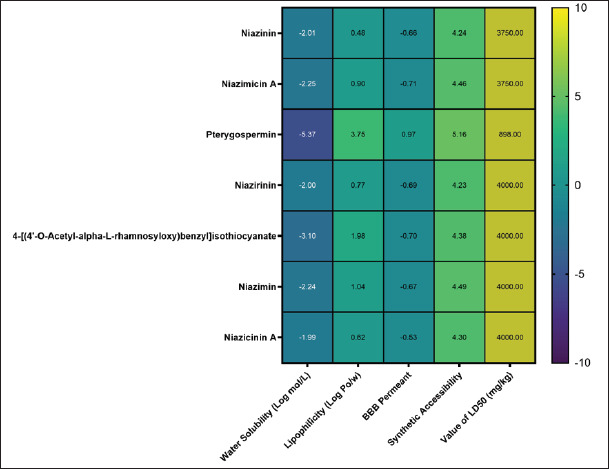
Heatmap displaying the ADMET properties of seven selected compounds from *M. oleifera*.

The ADMET characteristics of seven chosen *M. oleifera* compounds are revealed in this investigation (Fig. 2). The results demonstrated that Lipinski had neither violated nor approved of the drug-likeness characteristics of 4-[(4’-O-Acetyl-alpha-L-rhamnosyloxy)benzyl] isothiocyanate, Niazinin, Niazicinin A, Pterygospermin, and Niazirinin. All of the substances revealed an appropriate range of lipophilic and hydrophilic characteristics. Pterygospermin and Niazicinin A, on the other hand, demonstrated the highest levels of lipophilicity and water solubility, at 3.75 (Log P_0/w_) and −1.99 (Log mol/l), respectively. Additionally, seven selected compounds showed high GI absorption. The ADME prediction revealed that most of the selected compounds showed negative values of the BBB, which predicted they would not be able to pass through the BBB.

The current investigation shows that niazinin, niazimicin A, and pterygospermin are not P-gp substrates, indicating that their intestinal absorption and bioavailability are likely to be promising [12]. This finding showed that selected compounds of *M. oleifera* are in an acceptable range of LD50 value, which is category III with LD50 values of 500–5,000 mg/kg. In addition, pterygospermin showed the lowest LD50 values of 898 mg/kg, and niazirinin, 4-[(4’-O-acetyl-alpha-L-rhamnosyloxy)benzyl]isothiocyanate, niazimin, and niazicinin A have the highest LD50 values of 4,000 mg/kg. This present study revealed that 7 selected compounds are predicted to be safe for oral administration and do not have lethal effects.

### Molecular docking analysis of M. oleifera compounds

**Figure 3.**
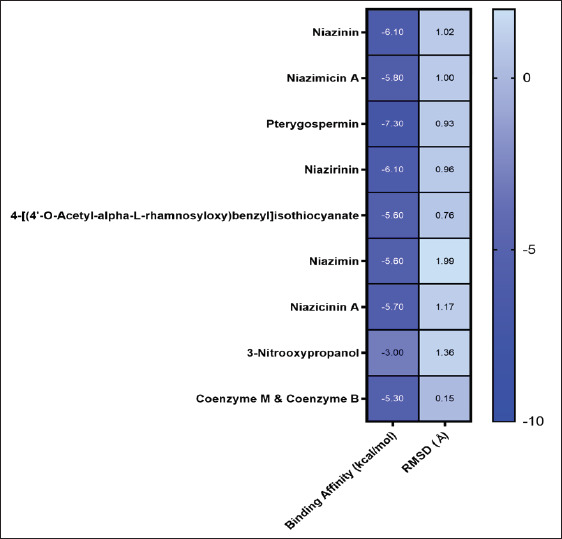
Heatmap displaying the binding affinity of seven selected compounds from *M. oleifera*.

This finding revealed that methyl-CoM and CoB, as the native substrates/ligands of MCR, showed a binding affinity of −5.3 kcal/mol (Fig. 3). In this present study, seven selected compounds of *M. oleifera* showed higher binding affinity compared to the native ligands (methyl-CoM and CoB), which are niazinin, niazimicin A, pterygospermin, niazirinin, 4-[(4’-O-acetyl-alpha-L-rhamnosyloxy)benzyl] isothiocyanate, niazimin, and niazicinin A, with −5.7, −6.1, −5.8, −7.3, −6.1, −5.6, −5.6, and −5.7 kcal/mol, respectively (Fig. 3). Based on that result, the selected compounds from *M. oleifera* are promising strong and potential inhibitors for methane production. On the other hand, the comparative ligand (3-NOP) showed weak inhibition against MCR with a binding affinity of −3.0 kcal/mol.

Additionally, the Discovery Studio Visualizer, which is shown in Table 3, can be used to visualize the interaction between ligands and macromolecular residues on receptors (MCR). The molecular interactions of pterygospermin with the targeted receptors included several different key residues. This study demonstrates that pterygospermin engages several key residues and a diverse bond network, including Pi-sulfur (Phe443), Pi-alkyl (Val482, Leu320, and Met324), Pi-Pi stacking (Phe330), and van der Waals interactions (Tyr333 and Ser325), all of which support hydrogen bonds and contribute to the stability of the complex [13].

**Table 3.** Visualization of residue interaction of phytochemical against MCR.

Niazinin	
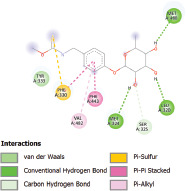	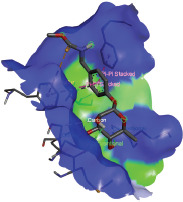
Niazimicin A	
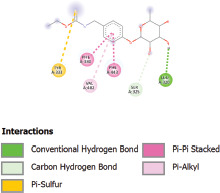	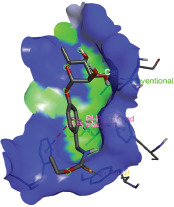
Pterygospermin	
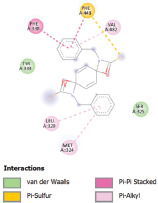	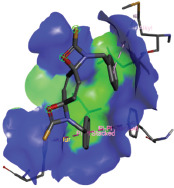
Niazirinin	
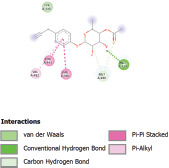	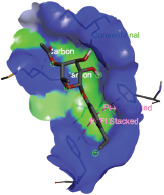
4-[(4'-O-Acetyl-alpha-L-rhamnosyloxy)benzyl]isothiocyanate	
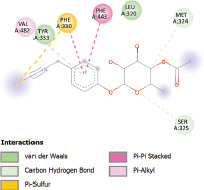	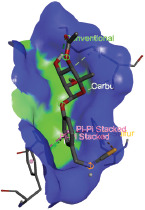
Niazimin	
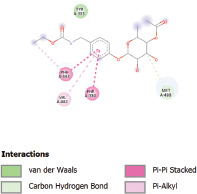	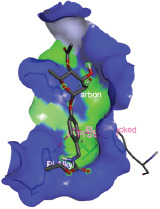
Niazicinin A	
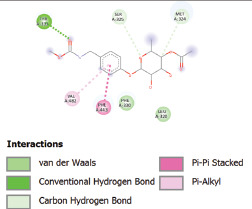	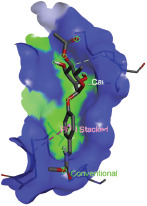
3-NOP	
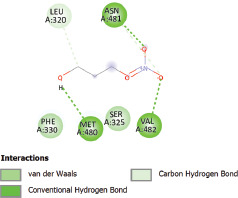	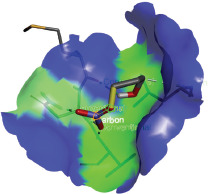

## Discussion

3-NOP has been demonstrated to be a successful feed addition for mitigating enteric methane, and it is advised that ruminants be continually fed it by incorporating it into their daily ration [14]. On the other hand, MCR is the enzyme involved in methane production in methanogenic Archaea. The substrate methyl CoM can enter the enzyme’s active sites via a narrow path that is locked once the second substrate, CoB, has bound. In this state, the MCR enzyme contains bound CoM and CoB, and its reactivation is limited to partial recovery through enzymatic reduction [15]. While 3-NOP effectively reduces methane emissions [14], phytochemicals from *M. oleifera* offer a promising alternative. The comparison between *M. oleifera* phytochemicals and 3-NOP in methane mitigation can be further expanded by investigating their potential role as methane inhibitors. Certain phytochemicals of *M. oleifera* may exert their effects through a competitive inhibition mechanism against the MCR enzyme. By competing with the natural substrate of MCR, the phytochemicals of *M. oleifera* could interfere with the final step of methanogenesis, thereby reducing methane production. In addition, phytochemicals offer a sustainable alternative to synthetic inhibitors while also providing additional benefits, such as antimicrobial properties, improved animal health, and enhanced feed efficiency [10].

It has been demonstrated that incorporating *M. oleifera* into the diet can control microbial metabolism and reduce methane emissions. In *in vitro* experiments, the inclusion of *M. oleifera* leaf and seed extracts significantly reduced rumen emissions by actively modulating the rumen microbiome. These methane-reducing effects are likely driven by phytochemicals that interact with rumen microbes, altering fermentation patterns and inhibiting methanogen activity [4]. Based on the previous study, the present study employs molecular docking to explore the potential of *M. oleifera* phytochemicals as effective methane inhibitors. This approach to identifying specific compounds capable of targeting MCR provides insights into their mode of action and potential application as natural feed additives for enteric methane reduction.

Drug-likeness is generally characterized by specific physicochemical parameters, including a molecular weight below 500 Daltons (Da), no more than five hydrogen bond donors, fewer than ten hydrogen bond acceptors, a lipophilicity (Log P) value not exceeding five, and a molar refractivity within the range of 40–130 [16]. Drug design focuses significantly on Lipinski’s rule of five, and it has been noted that compounds that violate any of these guidelines are likely to have low permeability or poor absorption [17]. The molecules of drugs intended for GI skin penetration should be relatively small, lipophilic, and electrically charge-free. The molar mass must be less than 500 Da in order to penetrate widely through the GI skin [18]. Hydrogen bonds are essential for molecular recognition, drug partitioning, structural stability, enzyme catalysis, and permeability in biochemistry [19]. Additionally, one crucial chemical feature for predicting the oral bioavailability of small drug candidates is the number of hydrogen bond donors and acceptors. Higher hydrogen bond donors or acceptors, however, can have an adverse effect on the drug’s permeability and membrane partition [20].

To be effective, a drug needs not only the desired bioactivity but also favorable pharmacokinetics, which requires a careful balance between its Log P and hydrophilicity. Log P, often measured by the Log P value, reflects how well a compound distributes between hydrophobic and hydrophilic phases [21]. In addition, the higher Log P value indicates that the molecule is more hydrophobic, making it more likely to be retained in the cell membrane’s lipid bilayer. It can lead to a broader distribution of the compound, which might reduce its selectivity for the target [22]. Conversely, the negative value of Log P could not pass through the lipid bilayer membrane, which caused a decrease in compound permeability [23]. Molar refractivity is a steric factor that reveals how small molecules interact spatially within biological environments [24]. Drug solutions’ molar refraction and polarizability in water are crucial concepts in pharmaceutical and medicinal chemistry because most biological reactions occur in aqueous environments [25].

Synthesized or isolated compounds were screened against the *in vivo* model to identify potential drugs. However, this method suffered due to the need to screen numerous quantities of molecules, making it labor-intensive. The quantity of active chemicals found was influenced by interactions with the target as well as additional elements like absorption, distribution, and metabolism [26]. Moreover, drug research and discovery require significant investments of energy, resources, and time. Advances in combinatorial chemistry and high-throughput screening have contributed significantly to the number of substances for which early data on ADMET properties are required [27].

Lipophilic molecules are more likely to adopt transcellular transport, while small hydrophobic molecules tend to favor paracellular transport. Other important permeation mechanisms include endocytosis, active transport, and carrier-mediated diffusion in addition to passive diffusion [26]. Based on solubility, Log P, and GI absorption, the ADME characteristics of each molecule indicate that the majority of its constituents have drug-like qualities, with the majority reaching the optimum absorption rate [16]. In addition, seven selected compounds of *M. oleifera*, with a synthetic accessibility score range of 4 and 5 out of 10, fall into the medium range for synthesis, suggesting that it is feasible but may require optimization to reduce production costs. The synthetic process could be streamlined by identifying and developing efficient synthetic pathways for extracted compounds, enabling its large-scale production. Predicting BBB penetration is essential for determining whether a drug can cross into the brain, which is crucial for reducing side effects, lowering toxicity, or enhancing drug efficacy. While pterygospermin has shown a positive value for BBB penetration, indicating it can cross the barrier, the value is less than 1, implying that it may be inactive in the central nervous system [28].

P-gp serves as a primary obstacle to the effective delivery of drugs, as it actively pumps toxins and foreign substances out of cells [29]. Moreover, the acute toxicity of a compound needs to be assessed to prevent long-term lethal effects. The median LD50, which is the single oral dose anticipated to result in death in 50% of test animals, is the term frequently used to characterize acute oral toxicity [30]. The following are the categories of toxicity: compounds with LD50 values ≤50 mg/kg are included in Category I; those with LD50 values >50 mg/kg but <500 mg/kg are included in Category II; those with LD50 values >500 mg/kg but <5,000 mg/kg are included in Category III; and those with LD50 values >5,000 mg/kg are included in Category IV [31]. Oral LD50 values between 0 and 500 mg/kg (Categories I and II) are regarded as extremely toxic, while LD50 values over 500 mg/kg are classified as having low toxicity [32].

ADMET analysis plays a crucial role in bridging computational predictions with practical implications in livestock research. ADMET screening supports prioritizing drugs with high bioavailability and few side effects by forecasting important pharmacokinetic parameters such as absorption, metabolism, and toxicity. This method enhances the use of resources for *in vivo* research [33]. This study suggested that seven selected compounds of *M. oleifera* with high predicted intestinal absorption and low hepatic metabolism are more likely to exhibit desirable pharmacokinetics in livestock, making them strong candidates for further experimental validation. Furthermore, ADMET data refines study designs by informing dosage strategies, metabolic pathways, and safety thresholds, reducing the reliance on trial-and-error approaches. This predictive framework ensures that only the most promising compounds proceed to animal trials, improving research efficiency and cost-effectiveness [34]. Validating ADMET predictions through *in vivo* studies strengthens the correlation between *in silico* models and biological responses, enhancing the reliability of computational methods for future compound screening.

MCR is the enzyme responsible for methane production in microbes. MCR converts methyl-CoM and CoB into CH_4_ and the heterodisulfide of CoM and CoB. Annually, approximately 109 tons of CH_4_ are produced, which escapes into the atmosphere and contributes significantly as a greenhouse gas. This makes understanding methane production crucial for environmental studies [15]. The phosphate group of CoB interacts with MCR residues positioned halfway down the channel, specifically with its thiol group from the nickel. The methyl-CoM binding site is located further inside the enzyme, suggesting that this substrate is essential for productive chemical reactions. This has been confirmed by steady-state and single-turnover kinetic studies [35]. It is hypothesized that when both substrates are bound in the active site, CoB induces a conformational rearrangement that positions methyl-CoM in closer proximity to the nickel center, thereby facilitating the cleavage of the carbon-sulfur bond [36].

Predicting the ligand’s orientation, location, and conformation within the binding site, as well as determining its binding affinity, are steps in the docking process. Identifying the binding site in advance significantly improves docking efficiency. Docking must determine the most advantageous binding mode within the protein’s binding pocket or active site to rank docked ligands appropriately. This requires determining the grid box or central coordinates where ligand-protein interactions occur within the active site [37]. Additionally, [38] revealed that the ligand-receptor interactions are influenced by the binding affinity value, where a lower value of binding affinity indicates a more stable binding between the ligand and receptor. The energy needed for the ligand to connect with the receptor (MCR) binding site is represented by the binding affinity. Several compounds from *M. oleifera* (niazinin, niazimicin A, niazirinin, 4-[(4’-O-Acetyl-alpha-L-rhamnosyloxy)benzyl] isothiocyanate, niazimin, niazicinin) demonstrated favorable ADMET properties, indicating that these compounds are likely to have good oral bioavailability and low toxicity. Furthermore, a ligand location that more closely resembles the natural ligand shape is indicated by a smaller RMSD value. Greater precision in the results is indicated by an RMSD of less than 2 Å, which indicates a reduced calculation error (Fig. 3) [39]. However, not all of them showed high binding affinity compared to pterygospermin (−7.3 kcal/mol). According to this, pterygospermin showed a more effective and stronger inhibition of methane generation, which makes it a viable option for additional research into methane mitigation techniques.

The active site is encircled by Phe330, Tyr333, and Phe443, which are situated close to aromatic and hydrophobic residues [15]. Among all methyl-CoM reductases, these amino acids are conserved [40]. Furthermore, CoB’s thiol group interacts with Val482’s main chain peptide nitrogen [15]. During the methane generation process, CoB is released, and the activated methyl group absorbs a hydrogen atom. The thiol group of CoB and the peptide nitrogens of Val482 interact in the MCR_ox1-silent_ structure to facilitate proton cleavage and the creation of a thiolate anion. Intermolecular forces such as hydrogen bonds, van der Waals contacts, and electrostatic interactions are crucial for determining the stability and bond energy values of molecular interactions [39]. Methane production inhibitors’ biological activity is influenced by these forces, which are especially important when interacting with important residues in the targeted receptor’s (MCR) active site (Table 3). This explains why pterygospermin exhibits a higher binding affinity than the native ligands, CoM and CoB, effectively competing with them to inhibit MCR activity. By forming strong intermolecular interactions within the MCR active site, pterygospermin disrupts the final step of methane synthesis in methanogenic archaea, thereby enhancing its inhibitory potential [41].

Pterygospermin, with its strong binding affinity and promising ADMET properties, offers a potential alternative that could outperform existing solutions, particularly in terms of sustainability and cost-effectiveness.

## Conclusion

The study has identified seven selected compounds derived exclusively from the seeds and leaves of *M. oleifera*, such as niazinin, niazimicin A, pterygospermin, niazirinin, 4-[(4’-O-Acetyl-alpha-L-rhamnosyloxy)benzyl]isothiocyanate, niazimin, and niazicinin A, all of which exhibit promising drug-likeness and favorable ADMET properties. Molecular docking analysis revealed that these seven compounds exhibit higher binding affinities compared to the native ligands, CoM and CoB, indicating strong potential as competitive inhibitors of MCR. Pterygospermin demonstrated the highest binding affinity to the MCR enzyme of −7.3 kcal/mol. Pterygospermin binding interactions with the enzyme’s active site include Pi-sulfur (Phe443), Pi-alkyl (Val482, Leu320, and Met324), Pi-Pi stacked (Phe330), and van der Waals interactions (Tyr333 and Ser325). Further experimental validation is needed, particularly through *in vitro* studies, to confirm the efficacy of pterygospermin as a feed additive for ruminants. Moreover, these findings could facilitate the adoption of plant-based solutions, offering a sustainable, eco-friendly approach to reducing the global greenhouse gas emissions of the livestock industry.

## List of abbreviations

ADMET, absorption, distribution, metabolism, excretion, and toxicology

BBB, blood-brain barrier

CoB, coenzyme B

CoM, coenzyme M

GI, gastrointestinal

LD50, lethal dose 50

MCR, methyl coenzyme M reductase

NOP, Nitrooxypropanol

PDB, Protein Data Bank

RMSD, root mean square deviation

SDF, Structured data file.
